# Comprehensive characterization of the alternative splicing landscape in ovarian cancer reveals novel events associated with tumor-immune microenvironment

**DOI:** 10.1042/BSR20212090

**Published:** 2022-02-09

**Authors:** Dan Sun, Xingping Zhao, Yang Yu, Waixing Li, Pan Gu, Zhifu Zhi, Dabao Xu

**Affiliations:** 1 Department of Gynecology, Third Xiangya Hospital of Central South University, Changsha 410013, Hunan, China; 2 Department of Gynecology and Obstetrics, The First Affiliated Hospital of Guangxi Medical University, Nanning 530021, Guangxi, China

**Keywords:** alternative splicing, genome-wide analysis, ovarian cancer, prognosis, tumor-immune microenvironment

## Abstract

**Background:** Ovarian cancer (OV) is a serious threat to women’s health. Immunotherapy is a new approach. Alternative splicing (AS) of messenger RNA (mRNA) and its regulation are highly relevant for understanding every cancer hallmark and may offer a broadened target space.

**Methods:** We downloaded the clinical information and mRNA expression profiles of 587 tumor tissues from The Cancer Genome Atlas (TCGA) database. We constructed a risk score model to predict the prognosis of OV patients. The association between AS-based clusters and tumor-immune microenvironment features was further explored. The ESTIMATE algorithm was also carried out on each OV sample depending on the risk score groups. A total of three immune checkpoint genes that have a significant correlation with risk scores were screened.

**Results:** The AS-events were a reliable and stable independent risk predictor in the OV cohort. Patients in the high-risk score group had a poor prognosis (*P*<0.001). Mast cells activated, NK cells resting, and Neutrophils positively correlated with the risk score. The number of Macrophages M1 was also more numerous in the low-risk score group (*P*<0.05). Checkpoint genes *CD274*, *CTLA-4*, and *PDCD1LG2*, showed a negative correlation with the risk score of AS in OV.

**Conclusions:** The proposed AS signature is a promising biomarker for estimating overall survival (OS) in OV. The AS-events signature combined with tumor-immune microenvironment enabled a deeper understanding of the immune status of OV patients, and also provided new insights for exploring novel prognostic predictors and precise therapy methods.

## Introduction

Ovarian cancer (OV) is the leading cause of female reproductive system-related cancer deaths and the fifth leading cause of cancer-related deaths among all women in the United States [1]. Due to the intimate site of OV, there is no simple and reliable screening method, and the symptoms are inconspicuous. More than 70% of OV patients have already reached stage III or IV at the time of diagnosis, at which point the survival time and quality of life are not ideal. Moreover, this kind of tumor presents primary or secondary drug resistance to chemotherapy drugs [2,3], posing a serious threat to women’s health. The immune system plays an important role in the pathogenesis and disease progression of OV, so improving autoimmunity through immunotherapy can effectively prevent and control the occurrence of OV. The most dramatic development has been the approval of programmed cell death protein 1 (PD-1) by the Food and Drug Administration (FDA), which is used for the treatment of solid tumors, including OV, with high microsatellite instability and mismatch repair defects [4]. Although various immunotherapy methods have shown certain effects in the treatment of OV, the improvement of prognosis of advanced-stage OV by immunotherapy is still relatively limited, and new therapeutic targets and drugs need to be discovered through basic research and clinical studies.

Messenger RNAs (mRNAs) are the molecular templates for the synthesis of proteins. In eukaryotic organisms, the primary gene transcripts, premessenger RNA (pre-mRNA), are typically non-functional for protein synthesis until internal sequences (introns) are removed, and the remaining fragments (exons) are spliced together to generate mature mRNAs. In the late 1970s, it was firstly reported that viral pre-mRNAs and, soon after, those of the κ chain of immunoglobulins underwent a process called alternative splicing (AS) [5–8]. From the very beginning, AS was proposed as a powerful mechanism to amplify the genetic information of an essentially linear genome [9]. The splicing process and its regulation are highly relevant for understanding every hallmark of cancer, to the point that splicing alterations constitute another cancer hallmark [10–13].

Some researchers have suggested that evaluating the expression of circulating CA-125 and/or HE4-specific spliced forms could help to define a better assay for early epithelial ovarian cancer (EOC) detection [14–17]. Systematic transcriptome analyses of normal and ovarian cancer via RNA-Seq revealed that cancer-specific mRNA isoforms exist and could be used for improving diagnosis and/or selecting new targeted therapies [18]. The evaluation of splicing events in selected genes has also been used better to predict the prognosis of EOC patients [19]. Using the top 20 survival-associated splicing events, it was demonstrated that they could predict patients’ survival with an area under the curve (AUC) of 0.93 in receiver operating characteristic (ROC) analyses [19]. Similarly, AS events have been used to predict chemoresistance in OV [20], overall suggesting that AS deregulation could be explored as a biomarker at different stages of EOC disease. Although progress made to expand the immunotherapy target space using tumor-specific mRNA processing events has been significant, much work is needed [21].

To the best of our knowledge, there is a scarcity of studies providing a comprehensive analysis of AS and its clinical significance in a tumor-immune environment of OV. In the present study, based on the newest RNA sequencing data of The Cancer Genome Atlas ovarian serous cystadenocarcinoma (TCGA OV) cohort, we conducted systematic profiling of genome-wide AS events in OV and identified OV-related AS events. In addition, the integration of clinical information and RNA-Seq data provided an insight into the prognostic value of AS events. We further explored the association between AS-based clusters and tumor-immune microenvironment features. A total of three immune checkpoint genes that have a significant correlation with risk scores had been screened. These findings help us better assess the prognosis of OV patients and provide assistance for immunotherapy.

## Methods

### Data collection and processing

The mRNA expression profiles and corresponding clinical data for the OV cohort were downloaded from the TCGA database (July 2021, https://portal.gdc.cancer.gov/). The AS event data for OV were obtained from the MD Anderson Center (https://bioinformatics.mdanderson.org/TCGASpliceSeq/) [22]. We fully assessed the availability of clinical information. A few patients were excluded because they met the following criteria: lack of complete clinical features (e.g., age, grade, International Federation of Gynecology and Obstetrics [FIGO] stage, and survival data). The percent spliced in (PSI) value was used to quantify each AS event, which is the ratio of normalized reads indicating the presence of a transcript element versus the total normalized reads for that event, with a rating from 0 to 1. The equation was as follows: PSI = splice in/splice in+splice out. We screened the AS data for PSI value > 0.75, representing the association of gene expression and AS events. We merged the gene expression and clinical profiles using the software Perl v5.30.0 (https://www.perl.org/get.html), thereby establishing genomics and clinical database for further research. A total of 410 patients with complete AS event data and clinical data were included in our analysis. The clinical features of the participants are summarized in Table 1.

**Table 1:** Demographic and clinical characteristics for 410 OV patients

Characteristics	Count	Percentage (%)
**Age (mean ± SD)**	59 (61.88 ± 11.43)	
≥65 (y)	280	68.29
<65 (y)	130	31.71
**Follow-up (mean ± SD) (y)**	3.28 ± 2.61	
**Status**		
Alive	157	38.29
Dead	253	61.71
**Histological type**		
Cystic, mucinous, and serous neoplasms	410	100.00
**FIGO stage**		
I	1	0.24
II	24	5.85
III	319	77.80
IV	64	15.61
Unknown	2	0.49
Grade		
G1	1	0.24
G2	46	11.22
G3	353	86.10
Unknown	10	2.44

Abbreviation: SD, standard deviation.

### Screening for prognostic AS events in OV

The database of TCGA SpliceSeq is based on TCGA RNA-Seq data. It includes seven types of selective splicing events, Alternate Acceptor site (AA), Alternate Donor site (AD), Alternate Promoter (AP), Alternate Terminator (AT), Exon Skip (ES), Mutually Exclusive Exons (ME), and Retained Intron (RI). We analyzed the distributions of all encoded genes using the UpSet.R package in each of the seven different types of AS events and survival-related AS events in OV.

### Construction of prognostic models and survival analysis

Different AS events in genes led to diversity in outcomes, and changes in gene expression affected survival time. To further understand the prognostic value of AS events in OV patients, univariate Cox regression analysis was performed with R package ‘survival’ to determine survival-related differentially expressed alternative splicing (DEAS) events, including overall survival (OS)-related DEAS events. Next, the least absolute shrinkage and selection operator (LASSO) regression was applied to identify the final elimination of potential predictors with non-zero coefficients using the R package ‘glmnet,’ which can avoid model overfitting to obtain a better fitting model. Furthermore, according to the results of LASSO Cox regression, predictive models were constructed using multivariate Cox regression analysis. Based on PSI values and multivariate Cox analysis, we calculated the risk scores of each participant and obtained the corresponding coefficients, respectively. The following formula can obtain the risk score:

$$\text{Risk score }=\;\sum_{\left(i=0\right)}^{n}\text{ PSI }\times \;\beta_{\text{i}}$$

where β is the regression coefficient of the AS events. A total of 410 OV patients were divided into high- and low-risk groups bound by the median risk score, and Kaplan–Meier survival analysis was performed to determine whether they had completely different prognoses. Furthermore, ROC curves of 1, 3, and 5 years were generated to show the discrimination of predictive signatures using the survival ROC package in R [23].

### Establishment and validation of a predictive nomogram

All clinical factors, including risk score, age, FIGO stage, and grade, were incorporated to construct a nomogram to evaluate the probability of 1-, 3-, and 5-OS of OV in the entire set. Validation of the nomogram was evaluated by the calibration plot with the ‘rms’ package. The calibration curve of the nomogram was plotted to assess the nomogram predicted probabilities against the actual rates.

### Immunescore estimate, immune cell infiltrating proportion inference

Normalized RNA expression data were used to infer the immunescore by the estimate package [24] and quantify the infiltrating proportions of 22 types of immune cells by the ‘CIBERSORT’ package [25]. The infiltrating percentage of 22 types of immune cells was equal to 100%. Single-sample Gene Set Enrichment Analysis (ssGSEA) was used to quantify and classify the immunity stage based on immune-related gene (IRG) sets [26]. Next, the differences of 16 hub immune checkpoints among the high- and low-risk groups were analyzed, and the correlations among the 6 most important immune checkpoint genes (*CD274*, *PDCD1*, *PDCD1LG2*, *CTLA4*, *HAVCR2*, *IDO1*) and risk score of AS events were further conducted. Relationships between the risk score of AS events and the number of immune cells were also evaluated by R.

### Analysis of the relationship between stromal/immune scores of OV tumor-immune microenvironment

The ESTIMATE algorithm was applied to analyze the Stromal Score, Immune Score, ESTIMATE Score, and Tumor Purity based on transcriptome profiles of OV to testify the effect of ssGSEA grouping.

### Statistical analysis

Statistical analyses were performed using R version 4.1.0 (http://www.cran.r-project.org). The *P-*values were two-sided and *P*<0.05 was considered statistically significant. If appropriate, the *P-*values were adjusted using the R package ‘qvalue.’

## Results

### Overview of AS events in TCGA OV cohort

**Figure 1:**
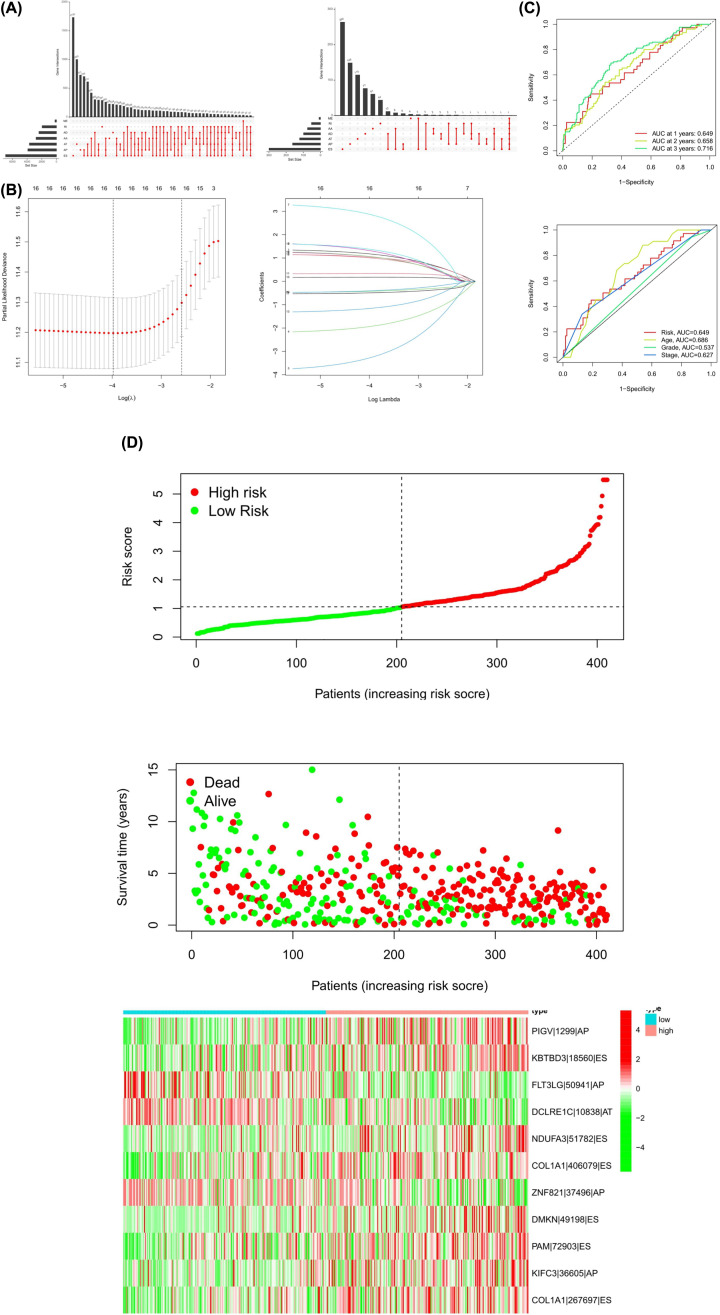
Identification and prognostic value of the AS markers in OV (**A**) UpSet plot of intersections and aggregates among diverse types of AS and survival-associated AS events in OV. (**B**) LASSO coefficient profiles of survival-associated AS events and ten-times cross-validation for tuning parameter selection in the LASSO model. (**C**) ROC curve in the predicted groups (high- and low-risk groups) by the signature of the 11-AS event in the OV cohort. (**D**) Risk score distribution of 11-AS events signature in the TCGA cohort including risk scores, survival status, and heatmap of the 11-AS events PSI profiles.

A total of 587 clinical samples and 412 AS samples were downloaded from the TCGA database. Finally, a total of 410 OV patients were identified, and the baseline characteristics of these patients are summarized in Table 1. The mRNA splicing data enrolled in the present study contains 48049 AS events in 10212 genes, including 19251 Exon Skip (ES), 9689 Alternate Promoter (AP), 8453 Alternate Terminator (AT), 4006 Alternate Acceptor (AA), 3497 Alternate Donor (AD), 2946 Retained Intron (RI), and 207 Mutually Exclusive Exons (ME). Given the possibility of multiple splicing modes for a single gene, we created UpSet plots to analyze interactive sets of seven types of AS events quantitatively. As shown in Figure 1A, a single gene could have up to six different splicing modes, and most genes had more than one AS event. The ES was the most frequent splice type among the seven AS types (40%), followed by AP (20.1%) and AT (17.5%), and ME was the least (0.4%).

### Prognostic index models featured by AS events for OV

**Figure 2:**
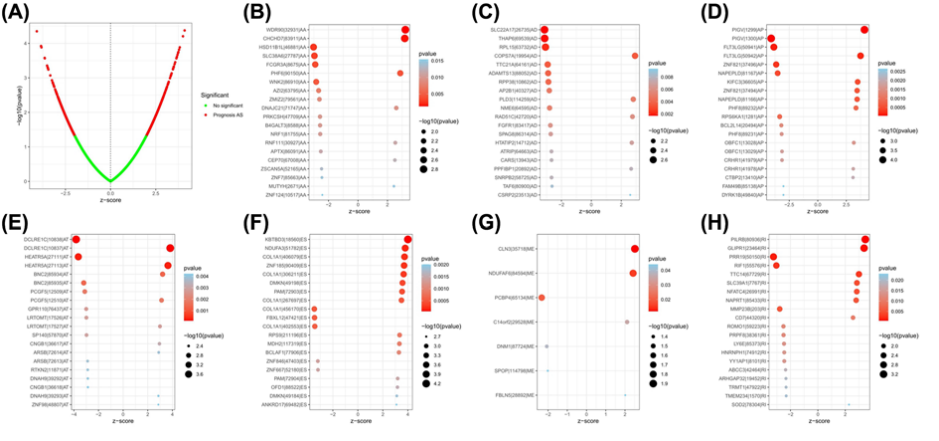
Forest plots analysis of survival-related AS events (**A**) The Volcano plot depicts the *P*-values from the univariate Cox analysis of 48049 AS events. (**B–H**) Forest plots of z-score of the top 20 significantly survival-related AS events for seven splicing types (ME has only seven events).

To explore the prognostic utility of AS signatures in OV, AS events associated with OS were identified by fitting univariate Cox proportional hazard regression models. Consequently, 1130 AS events were determined with adjusted *P*<0.05, including 530 high-risk survival-associated AS events (hazard ratio [HR] > 1) and 550 low-risk survival-associated AS events (HR < 1) (Figures 1A and 2). Figures 2B–H show the top 20 significant prognosis-associated AS events of the seven types. The UpSet plot was generated to visualize the intersecting sets between different genes and survival-associated AS events (Figure 1A), indicating that one gene might have more than one survival-associated AS event. Notably, the three highest frequency survival-associated AS events were still ES, AP, and AT in the OV cohort.

After conducting univariate regression analysis, LASSO regression was performed to select the optimal survival-related AS events to construct the prediction models to avoid model overfitting based on OS (Figure 1B). Finally, an 11-AS event signature was identified as a predictor of survival in OV through the Cox proportional hazards regression model (Table 2). Meanwhile, the risk scores of each OV patient were calculated, and all participants were divided into low- and high-risk groups bound by the median risk score. The distribution diagram shows the survival risk score (top), survival status of OV patients (middle), and clustering heatmap of the PSI levels of 11-AS markers (bottom). The horizontal axis indicates the patients’ order of risk scores from low to high (Figure 1D).

**Table 2:** Eleven-AS events associated with the OS of patients with OV

ID	Coefficient	HR	HR.95L	HR.95H	*P*-value
PIGV|1299|AP	1.4581	4.2978	1.8518	9.9750	0.0007
KBTBD3|18560|ES	1.1740	3.2348	1.0761	9.7235	0.0366
FLT3LG|50941|AP	−2.1388	0.1178	0.0347	0.4000	0.0006
DCLRE1C|10838|AT	−4.4337	0.0119	0.0011	0.1249	0.0002
NDUFA3|51782|ES	3.4926	32.8697	2.2322	484.0158	0.0109
COL1A1|406079|ES	2.3950	10.9686	1.6206	74.2367	0.0141
ZNF821|37496|AP	−1.4838	0.2268	0.0752	0.6835	0.0084
DMKN|49198|ES	1.8773	6.5356	2.0156	21.1914	0.0018
PAM|72903|ES	1.6082	4.9941	1.3106	19.0298	0.0185
KIFC3|36605|AP	1.2874	3.6234	1.4552	9.0219	0.0057
COL1A1|267697|ES	1.4661	4.3322	1.4409	13.0253	0.0090

**Figure 3:**
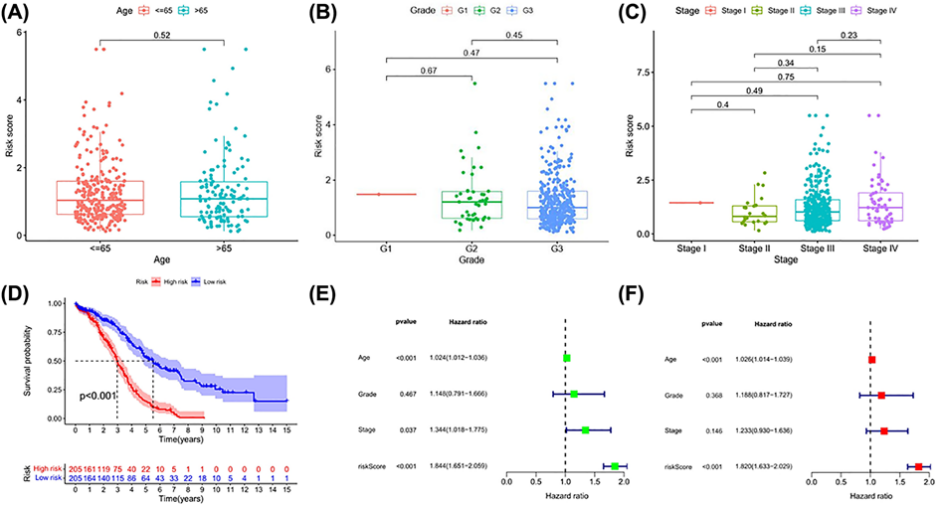
The risk score based on AS events are associated with survival and clinical parameters of OV patients (**A–C**) Differences in the risk score in age, grade, and FIGO stage groups. (**D**) The Kaplan–Meier survival curve of high-risk score group and low-risk score group. Univariate analysis (**E**) and multivariate analysis (**F**) of risk score and clinical characteristics that were simultaneously associated with OS. The bottom and top of the boxes are the 25th and 75th percentiles (interquartile range).

Kaplan–Meier curves and log-rank tests were plotted to explore the relationship between risk score and survival status. The survival probability of low-risk patients was higher than that of high-risk patients; in other words, high-risk patients had a higher mortality rate, as illustrated in Figure 3D (*P*<0.001). We then applied ROC analysis to compare the predictive power of these prognostic models, which showed a robust and significantly improved performance, with an ROC curve (AUC) in the third year greater than 0.700. Moreover, the AUC of risk score model predicting the 3-year survival rate was larger than that of the age, grade, and FIGO stage (Figure 1C).

### Construction and evaluation of the nomogram

**Figure 4:**
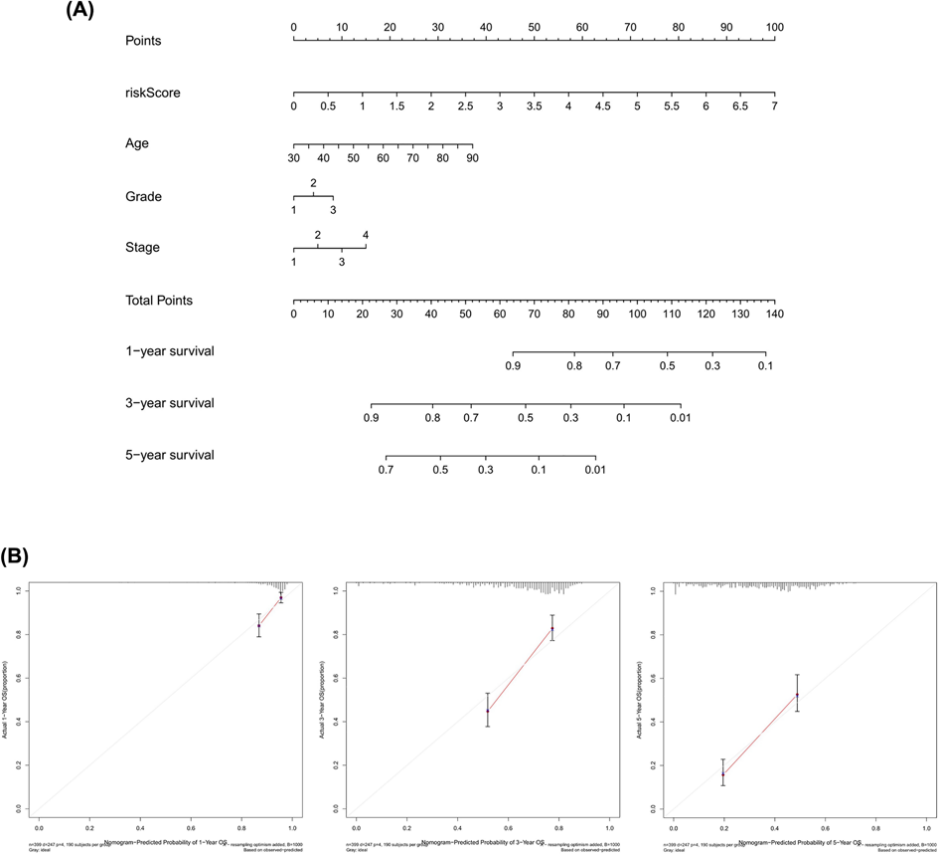
The establishment and validation of the nomogram (**A**) The nomogram consisted of age, gender, FIGO stage, and risk score was used to predict the 1-, 3-, and 5-year survival probability of OV patients. (**B**) Calibration plots of the AS-clinic nomograms are in agreement between nomogram-predicted and observed 1-, 3-, and 5-year outcomes of the OV cohort. Nomogram-predicted probability of survival is plotted on the x-axis; actual survival is plotted on the y-axis. The 45º dashed line represents the ideal performance. The red lines represent the actual performances of the model, and the figures from left to right show the 1-, 3-, and 5-year results.

Univariate and multivariate Cox regression methods were used and combined patient clinical characteristics (age, grade, and FIGO stage) to analyze whether the 11-AS event signatures could be an independent predictor of survival in patients with OV. The results showed that the risk score could still be used as a reliable and stable independent risk predictor in the OV cohort (*P*<0.001; Figure 3E,F). We constructed a predictive nomogram based on the multivariate analysis (Figure 4A) that included risk scores and clinical characteristics. As the red lines in the pictures are almost overlap with the 45º dashed lines, the calibration curve revealed that the predicted values are effective in the prediction of the 1-, 3-, and 5-year OS (Figure 4B). The results demonstrated that the risk score had satisfactory diagnostic ability and clinical characteristics (*P*<0.05). The 11 genes involved in the model construction by multivariate analysis are shown in Table 2. However, the risk score was found to have no differences in age, grade, and stage groups (Figure 3A–C, *P*≥0.05).

### The risk score and AS events are associated with the infiltration of immune cells in the OV tumor microenvironment

**Figure 5:**
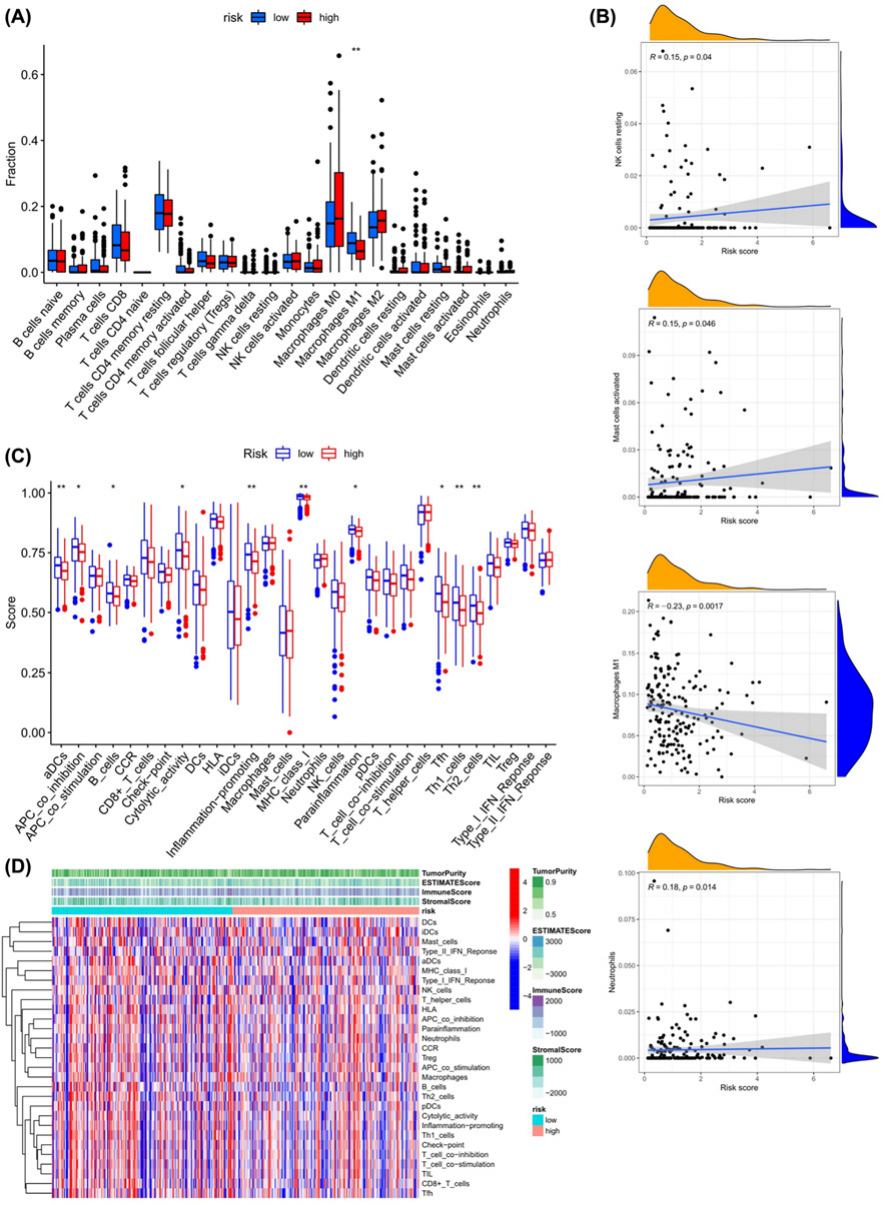
The relationship of the risk score and immune cells infiltrating in the OV tumor-immune microenvironment (**A**) The landscape of 22 types of infiltrating immune cells in the low-risk score and high-risk score groups. (**B**) The correlation analysis between immune cells and risk score. (**C**) The landscape of 29 types of infiltrating immune cells and immune function in two groups. The bottom and top of the boxes are the 25th and 75th percentiles (interquartile range). Blue: low risk, red: high risk. **P<0.01, *P<0.05. (**D**) The heatmap showed the difference in infiltrating immune cells in the two groups in an OV tumor-immune microenvironment.

First, the immune score in 29 types of infiltrating immune cells and immune function was assessed by the ssGSEA method [26]. Figure 5C,D show the immune score difference of each immune cell in the low-risk score group and high-risk score group. We further explored the impact of risk score on the infiltration of 22 types of immune cells in the tumor microenvironment (TME) by the CIBERSORT algorithm. The landscape of 22 types of immune cells infiltrating in the low-risk and high-risk scores groups is shown in Figure 5A. Correlation analysis results showed that three types of immune cells (Mast cells activated, NK cells resting, Neutrophils) had a positive correlation with the risk score, but Macrophages M1 showed a negative correlation with risk score (*P*<0.05) (Figure 5B).

### The risk score is associated with the key immune checkpoint genes in the tumor-immune microenvironment of OV

**Figure 6:**
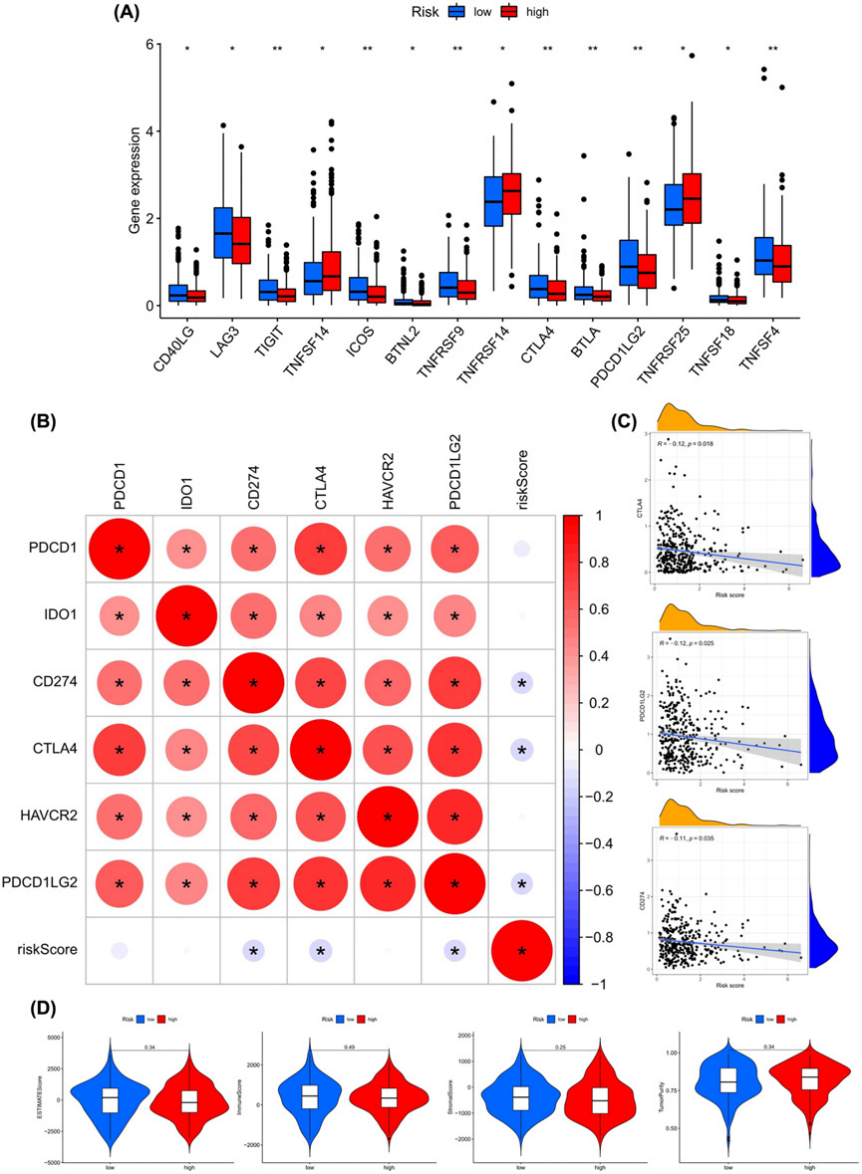
The key immune checkpoint genes and tumor-immune microenvironment are related to risk score in OV tumor-immune microenvironment (**A**) The landscape of 26 types of immune checkpoint genes in low-risk score and high-risk score groups. **P<0.01, *P<0.05. (**B**) The correlation of the risk score and the six most important checkpoint genes (*CD274, PDCD1, PDCD1LG2, CTLA4, HAVCR2, IDO1*). (**C**) The correlation analysis between immune cells and risk score. (**D**) Differences in Tumor Purity, ESTIMATE Score, Immune Score, and Stromal Score calculated between the two groups. The bottom and top of the boxes are the 25th and 75th percentiles (interquartile range). *: statistically significant; red: positive correlation, blue: negative correlation.

The difference in the expression level of 47 immune checkpoint genes in the low-risk and high-risk groups was assessed, and 14 genes had significant differences (Figure 6A). Next, R packages (limma, corrplot, ggpubr, ggExtra) were used for screening the risk score related to the six most important checkpoint genes (*CD274*, *PDCD1*, *PDCD1LG2*, *CTLA4*, *HAVCR2*, *IDO1*). With the absolute threshold value of *P*<0.001, three immune checkpoint genes *CTLA4*, *CD274*, and *PDCD1LG2* were identified (Figure 6B). The scatter plot displaying the correlation of those three genes and risk scores were plotted separately. Though three of the correlation coefficients did not reach 0.3, the scatter plot showed a negative correlation (Figure 6C).

### The stromal/immune scores in the OV microenvironment

The violin plot assessed the differences in Tumor Purity, ESTIMATE Score, Immune Score, and Stromal Score calculated using ESTIMATE algorithm between the two groups. No differences were found in the four groups (*P*≥0.05) (Figure 6D).

## Discussion

The current view is that a tumor is a disease of uncontrolled cell proliferation and one caused by a disorder of the microenvironment. The TME provides the material basis for tumor proliferation, metastasis, and invasion as the basis of tumor survival. The TME consists of complex components, including fibroblasts, immune cells, adipocytes, vascular endothelial cells, and extracellular matrix. Current therapies targeting the TME include targeting tumor neovascularization, TME stromal cells, and inhibiting tumor immune escape [27].

Immunotherapy has shown high clinical value in the treatment of OV in recent years. Both active and passive immunotherapy can exert a tumor-suppressive effect and improve the prognosis of patients [28–31]. However, the improvement of the prognosis of advanced OV by immunotherapy is still relatively limited. An important factor limiting the immunotherapeutic response and disease progression of OV is that the TME of OV is in an immunosuppressive state [32]. Our research showed that the AS-events signature combined with tumor-immune microenvironment allowed a deeper insight into the immune status of OV patients and also provided a new perspective for exploring novel prognostic predictors and precise therapy methods.

We discovered that Tumor Purity, ESTIMATE Score, Immune Score, and Stromal Score had no differences in high- and low-risk score groups. However, Macrophages M1 showed a negative correlation with a risk score, and the number of Macrophages M1 was also more numerous in the low-risk score group.

Macrophages are the most abundant immune cells in the TME, located in the tumor stromal solid region, and play a promoting or inhibiting role in tumor progression [33]. They are generally classified into two polarized phenotypes: M1 and M2 [34]. We found that the number of Macrophages M1 was also more numerous in the low-risk score group. The prognosis of low-risk score group is better than that of high-risk score group. So, we speculate that the aggregation of Macrophages M1 in TME may be related to the better prognosis of OV. Studies have shown that M1 is considered to have antitumor effects, while tumor-associated macrophages (TAMs) belong to the M2 macrophage phenotype [35]. It has been reported that macrophage polarization of OV affects tumor formation, growth, and metastasis through interaction with cancer cells [36]. A high M1/M2 ratio in ovarian tumors was associated with prolonged survival [37], while a low M1/M2 ratio was associated with poor OS [38]. The infiltration of M2 macrophages in ovarian serous carcinoma indicates a poor prognosis [39–40].

Inhibiting the transformation of TAMs to M2 type or the reversal of M2 phenotype to M1-type macrophage may restore macrophages’ immune activity and cytotoxicity, and inhibit angiogenesis and lymphangiogenesis, then subsequently achieve the purpose of inhibiting tumor growth, invasion, and metastasis. Experimental reports have shown that drugs regulating the transition from M2-type to M1-type can prolong the antitumor activity of macrophages [41]. We found that in the immune microenvironment of ovarian tumors, the higher the risk score of AS-events, the higher the content of M1, and the better the prognosis. Some studies have shown that drugs that regulate the polarization of macrophages can control the growth of OV cells. Paclitaxel is an antineoplastic drug used to treat OV and can reduce tumor growth by polarizing M2 into M1 macrophages in a TLR4-dependent manner [42]. It has been reported that the relationship between macrophage polarization and OV is affected by platinum. It was found that macrophages induced epithelial–mesenchymal transformation (EMT), but not in cisplatin-resistant cancer cells [43]. At present, there are some therapeutic drugs for TAMs that can be used in clinical and experimental treatment [44–46]. Furthermore, an abundance of work implicates the altered expression of genes encoding splicing factors in the widespread dysregulation of AS in cancer [47–49]. Several studies have shown that altered expression of such factors occurs in numerous different cancers and can be linked to malignant transformation [50–53]. How the change in AS leads to the transformation of M2 to M1, thus increasing the antitumor effect of tumor stromal cells, may become a new concept of immunotherapy.

Immune checkpoint inhibitors are the most widely used immunotherapy in cancer. The 2018 National Comprehensive Cancer Network (NCCN) Clinical Practice Guidelines for cervical cancer, endometrial cancer, and OV all recommend pemumumab for use with microsatellite instability-high (MSI-H) or different mismatch repair (dMMR) in recurrent cervical, endometrial, and ovarian cancer. This means that the era of immunotherapy in gynecological oncology has officially arrived. Immune checkpoint inhibitors have achieved good efficacy in targeted therapy of gynecological malignancies, but the overall remission rate is not high. A trial evaluated the efficacy of PD-1 inhibitors in 12 tumor types with dMMR found that 21% of patients achieved a complete response [54]. Wang et al. [55] indicated that PD-1 AS isoforms b should be considered a biomarker for clinical responsiveness to anti-PD-1/PD-L1 immunotherapy; isoform c had a prometastatic role and is a new potential target for colorectal cancer therapy. Both PD-1Deltaex2 and PD-1Deltaex3 are generated by AS where exons 2 and 3 are respectively spliced out [56]. A parallel increase in the expression of PD-1Deltaex3 and flPD-1 upon activation suggests an important interplay between the putative soluble PD-1 and flPD-1 possibly involved in maintaining peripheral self-tolerance and prevention of autoimmunity [56]. Another article supported the significance of PD1 AS in celiac disease as a novel source for diagnostic and therapeutic targets [57]. During the development phase of current therapies based on membrane-bound CTLA-4, 2 alternatively spliced mRNA transcripts of CTLA-4 were identified, encoding a secretable isoform of sCTLA-4 [58–60], and in mice ligand-independent CTLA-4 [61]. Plasma and serum levels of sCTLA-4 are raised in several human diseases, and it could be relevant to immune regulatory processes in patients [62]. We found that checkpoint genes *CD274*, *CTLA-4*, and *PDCD1LG2* showed a negative correlation with the risk score of AS in OV. How AS influences the mechanism of tumor immunity and immunotherapy by changing immune checkpoints remains to be further studied.

The current study also has several limitations. The study is based on bioinformatics analysis, and there are no recruited cohorts for prognostic verification. The lack of data in normal tissues makes it impossible to predict the differentially expressed AS-event related genes in cancer and normal tissues.

## Conclusion

Our research mainly assessed the heterogeneity of tumor-infiltrating immune cells in OV TME and found that three immune checkpoint genes *CD274*, *CTLA-4*, and *PDCD1LG2*, showed negative correlations with risk scores. Also, the proposed clinical-immune signature is a promising biomarker for estimating OS in OV. The AS-events signature combined with tumor-immune microenvironment allowed a deeper understanding of the immune status of OV patients, and also provided new insights for exploring novel prognostic predictors and precise therapy methods.

## Data Availability

The original contributions presented in the study are included in the article/Supplementary Material. Further inquiries can be directed to the corresponding authors.

## Abbreviations

AS: alternative splicing
AUC: area under the curve
dMMR: different mismatch repair
EOC: epithelial ovarian cancer
FIGO: International Federation of Gynecology and Obstetrics
HR: hazard ratio
LASSO: least absolute shrinkage and selection operator
mRNA: messenger RNA
NCCN: National Comprehensive Cancer Network
OS: overall survival
OV: ovarian cancer
PD-1: programmed cell death protein 1
pre-mRNA: premessenger RNA
PSI: percent spliced in
ROC: receiver operating characteristic
ssGSEA: single-sample Gene Set Enrichment Analysis
TAM: tumor-associated macrophage
TCGA: The Cancer Genome Atlas
TME: tumor microenvironment
